# Design and validation of an intelligent wheelchair towards a clinically-functional outcome

**DOI:** 10.1186/1743-0003-10-58

**Published:** 2013-06-17

**Authors:** Patrice Boucher, Amin Atrash, Sousso Kelouwani, Wormser Honoré, Hai Nguyen, Julien Villemure, François Routhier, Paul Cohen, Louise Demers, Robert Forget, Joelle Pineau

**Affiliations:** 1 Department de Génie Electrique, Ecole Polytechnique de Montréal, Montréal, Canada; 2 School of Computer Science, University of Southern California, Los Angeles, USA; 3 Department de Génie Mécanique, Université du Québec à Trois-Rivières, Trois-Rivières, Canada; 4 Centre de réadaptation Lucie-Bruneau, Montréal, Canada; 5 School of Computer Science, McGill University, Montréal, Canada; 6 Department of Rehabilitation, Université Laval, Québec, Canada; 7 Ecole de readaptation, Universite de Montreal, Montréal, Canada

**Keywords:** Assistive robotics, Intelligent powered wheelchairs, Wheelchair skills test

## Abstract

**Background:**

Many people with mobility impairments, who require the use of powered wheelchairs, have difficulty completing basic maneuvering tasks during their activities of daily living (ADL). In order to provide assistance to this population, robotic and intelligent system technologies have been used to design an intelligent powered wheelchair (IPW). This paper provides a comprehensive overview of the design and validation of the IPW.

**Methods:**

The main contributions of this work are three-fold. First, we present a software architecture for robot navigation and control in constrained spaces. Second, we describe a decision-theoretic approach for achieving robust speech-based control of the intelligent wheelchair. Third, we present an evaluation protocol motivated by a meaningful clinical outcome, in the form of the Robotic Wheelchair Skills Test (RWST). This allows us to perform a thorough characterization of the performance and safety of the system, involving 17 test subjects (8 non-PW users, 9 regular PW users), 32 complete RWST sessions, 25 total hours of testing, and 9 kilometers of total running distance.

**Results:**

User tests with the RWST show that the navigation architecture reduced collisions by more than 60% compared to other recent intelligent wheelchair platforms. On the tasks of the RWST, we measured an average decrease of 4% in performance score and 3% in safety score (not statistically significant), compared to the scores obtained with conventional driving model. This analysis was performed with regular users that had over 6 years of wheelchair driving experience, compared to approximately one half-hour of training with the autonomous mode.

**Conclusions:**

The platform tested in these experiments is among the most experimentally validated robotic wheelchairs in realistic contexts. The results establish that proficient powered wheelchair users can achieve the same level of performance with the intelligent command mode, as with the conventional command mode.

## Background

There are over 4.3 million users of powered wheelchairs in the US alone [1]. It has been reported that 10% of powered wheelchair users experience serious difficulties with the standard operation of their wheelchair, in particular with steering and maneuvering tasks [2]. Furthermore, there are many other individuals who require mobility assistance, yet also have other conditions, such as visual or cognitive impairments, that hamper their ability to safely operate a powered wheelchair. The development of an intelligent powered wheelchair (IPW) offers a promising technology to increase independence of all those individuals.

Various prototypes of IPWs have been developed over the years. These feature a variety of robotic technologies. In this section, we review some of the most recent results, and refer the reader to an excellent overview for a more detailed survey [3]. One of the primary challenges of building an IPW is in acquiring sufficient information from the surrounding environment. In terms of onboard navigation sensors, most systems rely on standard distance sensors, such as sonar, IR, laser range-finding, or binocular vision for mapping, localization and obstacle avoidance [3-6]. Laser range-finders, which offer the best accuracy in terms of range measurements, were relatively rare until recently due to their high-cost and large form factor. However the technology has been improving in this area, making them a more viable option.

Many IPW systems aim to offer autonomous hands-free navigation services. To achieve this, a variety of navigation modes have been considered, including reactive control, autonomous manoeuvre execution, and autonomous point to point navigation. In the reactive navigation mode, the user is responsible for motion planning and execution with the help of a collision avoidance system [7-9]. This system does not require the knowledge of the environment prior to navigation. The reactive mode is suitable for users who are able to plan their routes and to manipulate the input devices. In the autonomous manoeuvre execution mode, a set of navigation maneuvers is designed for specific navigation tasks [10-12]: doorway traversal [13-15], corridor traversal [13,15,16], wall following [16,17], automatic docking [4,18] and person following [19]. In the autonomous point-to-point navigation mode, the user selects its destination pose in the map and supervises the navigation process. Given the destination pose, the navigation system is responsible for platform localisation, path planning and plan execution with local obstacle avoidance [20-24]. Safe navigation has also been achieved through artificial potential fields [25], or obstacle density histogram [26]. In general, the full literature on robot navigation could be leveraged for this component, though it is necessary to respect the constraint imposed by the domain. For example, classical methods based on pose error tracking often do not lead to smooth motion; Mazo [27], Gulati and Kuipers [28] have proposed methods that tend to produce graceful motions. In the work presented below, we focus on the first two levels of capabilities (reactive control and autonomous manoeuvre execution). These are sufficient for deployment in the Wheelchair Skills Test environment. The third level is currently implemented, but was not validated in the experiments described below, therefore it is not described.

A variety of input methods have been incorporated onboard IPWs, from the traditional joystick or single-switch interface, to speech recognition, and most recently brain-computer interface. In his survey, Simpson [3] argued that the onboard computer system (presumably equipped with AI components) could provide a form of safety net against input methods with low-bandwidth or poor accuracy. Efforts have been divided into two main directions. The first direction focuses on using standard joystick input, and enriching this information with embedded intelligent systems to improve safety and efficiency of navigation [12,29]. The second direction leverages non-traditional control interfaces such as voice-activation and brain-computer interface to obtain high-level commands that are then translated into fine motor control by the onboard navigation system [30,31]. Our work falls primarily in this second category.

While many intelligent wheelchair systems have been developed, very few have been the subject of substantial validation with the target population. The situation has improved in the last few years, with a number of systems undergoing formal testing. However the choice of task domains and evaluation metrics still primarily comes from the robotic field (e.g. quantifying navigation performance), rather than from the rehabilitation domain (e.g. quantifying skills and improved functional outcomes). A review of commonly found metrics is presented by Urdiales et al. [32] whereas a detailed evaluation procedure for an intelligent wheelchair is presented by Montesano et al. [15].

The primary contribution of this paper is to present a fully integrated IPW which has been demonstrated to achieve flexible and robust performance with the target population in a clinically relevant environment. Unlike many of its predecessors, the robotic system presented here is designed to fit on any of a number of commercial powered wheelchair platforms. It provides rich sensing and communication interfaces to ensure robust operation and control in a variety of environmental conditions, in particular in constrained spaces that pose particular challenges for standard steering. Our robotic system is also designed to be used by individuals with varying impairments.

The main algorithmic components developed for the autonomous control of the wheelchair include an autonomous navigation system and a voice-activated communication system. Both of these components feature state-of-the-art robotic techniques, deployed in a challenging indoor experimental context. The navigation system is particularly proficient at handling autonomous navigation in narrow spaces, such as passing through doorways, aligning to a wall, and parking in a corner; these are the types of maneuvers that are particularly challenging for many wheelchairs (WC) users. The interaction system is substantially more flexible than previous such interfaces, allowing robust speech-based commands using full vocabulary natural language. We leverage several machine learning techniques to achieve robust speech understanding, including grammatical parsing to reduce the observation space, Bayesian inference techniques to track the most likely commands, and planning in Markov decision processes to select appropriate responses, or clarification queries when necessary. The combination of these methods is shown to produce a reliable, flexible, and congenial user interface.

Finally, one of the major contributions of the work reported in this paper is the method and results based on a validated evaluation of individual’s performance with a wheelchair. We adopt an experimental procedure based on the Wheelchair Skills Test [33], which requires completion of a varied collection of skills that are relevant to everyday operation of a powered wheelchair. Our experiments involved eight able-bodied and nine disabled subjects, 32 completed Robotics Wheelchair Skills Test (RWST) sessions, 25 total hours of testing, and 9 kilometers of total running distance.

## Methods

In this section we provide an overview of the intelligent wheelchair platform, including the hardware components that compose the robot, and the software modules developed for autonomous navigation.

### System overview

**Figure 1:**
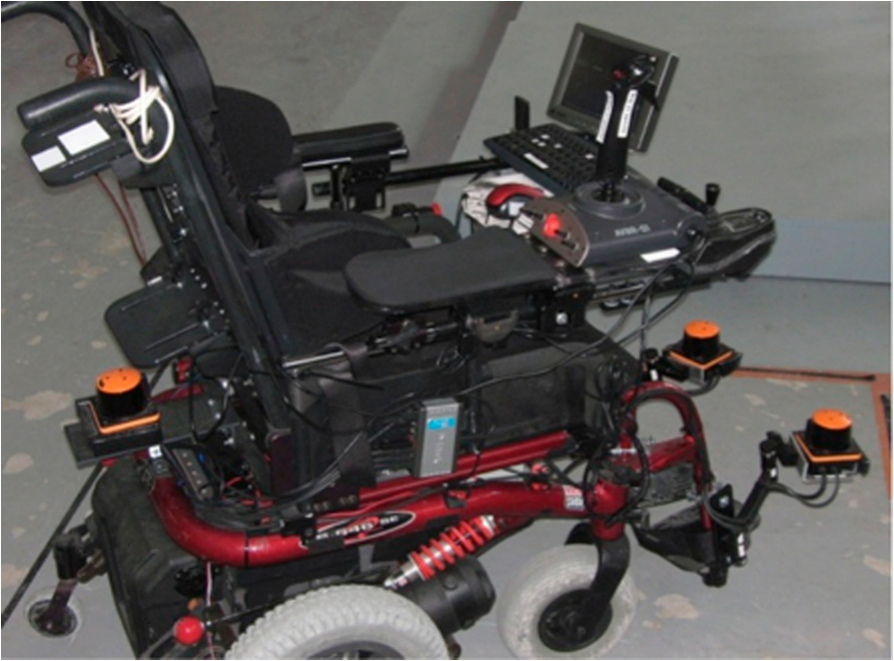
Intelligent power wheelchair prototype.

**Figure 2:**
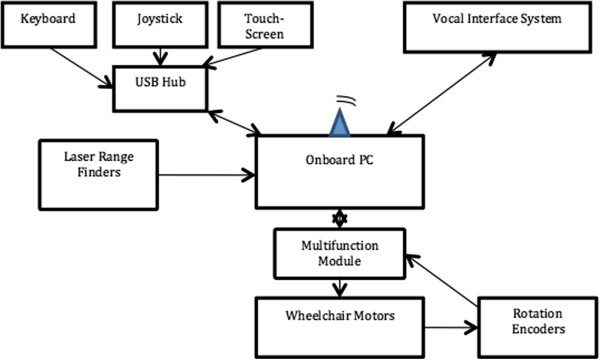
Hardware architecture.

The navigation system presented in this paper is based upon a modified commercial wheelchair (Sunrise Medical, Model Quickie S646) as illustrated in Figure 1. This differentially driven rear wheel platform is equipped with three planar laser range-finders for 360˚ environment coverage, two wheel-mounted rotation encoders, a joystick, a touch-screen, and a voice control interface. For experimental purposes, two additional input devices (i.e. a computer keyboard and a game joystick) have been added. All devices are connected to an on-board computer using a Universal Serial Bus (USB) concentrator. Figure 2 illustrates the wheelchair hardware architecture.

### Navigation system

The control architecture has the following characteristics:

Sensor-independent primitive behaviors, such as simple rectilinear motion, rotation, waypoints following, are implemented into generic classes that are used by subsequent decision modules;

An internal world representation includes the environment as well as the mobile platform. This representation avoids the problem of redundant analysis of raw measurements and offers sensor-independent sources of information to all decision modules, thus facilitating the design of high-level functionalities;

Complex tasks in restrained environments are executed though collaboration between high-level task-oriented modules and a motion assistance module [34].

#### Navigation system overview

**Figure 3:**
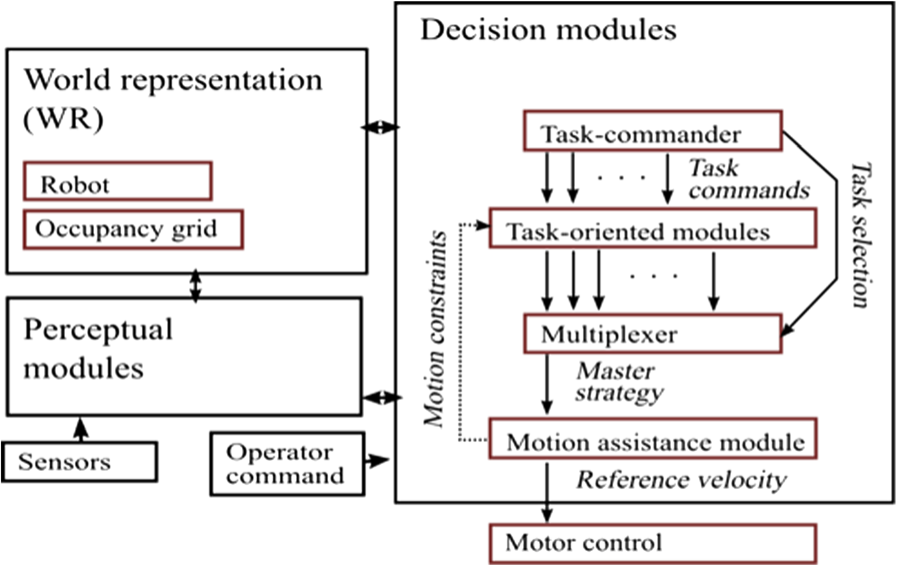
Control architecture of the semi-autonomous navigation system.

The control architecture, depicted at Figure 3, is composed of modules for perception, internal world representation, decision-making and multi-modal user communication. The internal representation serves as the information source for decision-making. It is updated in real-time using data from the perception modules, based upon raw sensor measurements linked with its current configuration.

#### Internal representation of state and perceptual modules

The internal representation, built and updated by the perceptual modules, represents a central base of information for reasoning and decision-making. It consists of a representation of the robot itself, including its sensors, and a dynamic occupancy map representing the local environment. The representation of the robot includes relevant characteristics of the wheelchair such as geometrical characteristics (geometry, weight, inertia, etc.), state (pose, speed, etc.) and on-board resources (sensors). The state component is updated in real time.

At this stage, the control approach does not rely on a learned representation of the environment, therefore no predefined navigation map is used. The use of a static map for high-level navigation specification and control will be added at a later stage of development, but is not necessary for the experiments we describe below. Rather, the environment is represented by a dynamic 2D occupancy grid. Each cell (labeled as free or occupied) is attributed a memory-decaying factor, in order to progressively eliminate the effects of skidding and moving objects. Map updating is performed as soon as new range data is acquired by the laser scanners and the on-the-fly evaluations simulations of planned actions is carried out in order to allow anticipative actions, as done in [35].

#### Decision modules

The decision modules are decomposed as a set of inter-dependent modules. Three main types of decision modules are involved: the first one for task selection, the second one for decomposition of high-level tasks and the third one for motion assistance (collision and deadlock avoidance). The task commander selects a task-oriented module within the second level according to the user command together with specific information related to the task (i.e. a riding distance, a distance from which a wall must be followed) and a driving profile. Each task-oriented module in the second level specializes in a specific high-level task and uses primitive behaviors that depend upon the user command and the operation context.

The motion assistance module at the bottom cooperates with task-oriented modules such as parking, door frame traversal and wall following. The cooperation takes the form of, first, choosing an optimal motion according to the motion strategy and objectives of the task-oriented modules and, secondly, informing them about the motion possibilities to help them adjust their future strategies.

Each task-oriented module has its own instantaneous motion strategy, consisting of a motion command (translation and rotation speeds), a target position, and an optional preferential rotation for bypassing obstacles. Based upon the motion command, a free-space index is calculated as the minimum distance between any point on the platform and the environment in the direction of the motion trajectory [26,34]. Whenever the free-space index is large enough (above a threshold), the motion trajectory is said to be admissible. However, the motion command magnitude is smoothly reduced in order to ensure safe navigation. In cases where the motion trajectory is not admissible and the preferential rotation for bypassing obstacles is set, an alternative motion is selected among a set of candidate fixed linear and angular speeds, among a range of possible directions (ahead, ahead-left, left, back-left, back, back-right, right, ahead-right). The selection process is based on the minimization of a cost function involving a motion objective, a target reaching objective and an obstacle avoidance objective.

#### Task-oriented modules

A number of task-oriented modules were implemented. The choice of these was motivated primarily by the clinical task domain used for our empirical validation. All of these maneuvers are useful for a much wider range of task domains; other maneuvers could be added for general navigation in other domains.

Task-oriented modules currently implemented and tested include:

Wall following (module M1)

Parking (module M2)

Door frame traversal (module M3)

Task-oriented modules use a set of primitive behaviors implemented by generic classes, allowing flexible configurations of primitive behaviors in order that they can be effectively adapted to every specific high-level task. The following primitive behaviors have been implemented:

Basic straight line displacement and simple rotation (C1)

Waypoint following (C2)

Straight line segment following (C3)

and are based on a set of human-inspired kinematic behaviors in [36] such as:

The linear speed decreases according to the angular error relative to the subsequent waypoint;

Linear and angular speeds vary according to future states in order to produce a continuous motion (anticipation);

Speeds are continuously adjusted in order to minimize execution duration while ensuring convergence toward waypoints;

Table 1 summarizes task-oriented modules and their corresponding primitives behaviors

**Figure 4:**
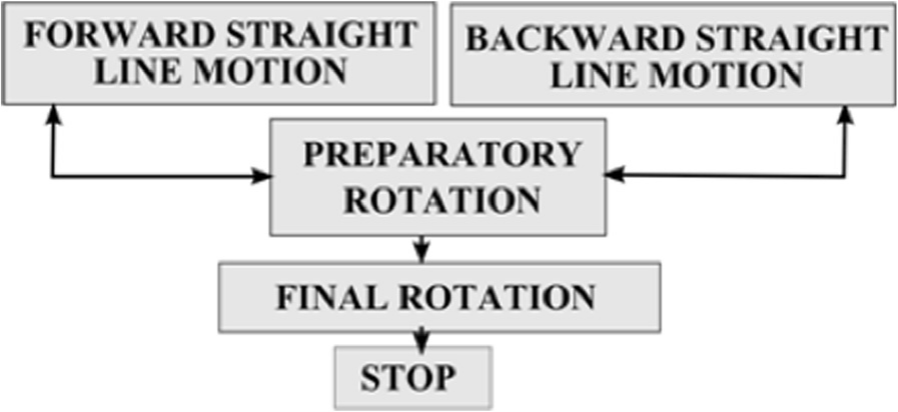
State machine defining the parking strategy.

1. Wall-Following Module (M1)

This agent is meant to reduce control efforts when the platform has to navigate close to lateral obstacles or walls. A straight line is built at each execution time according to the current lateral obstacles. Primitive C3 handles the straight-line displacements while primitive C1 is occasionally used to locally change the platform orientation due to changes in wall orientation.

2. Parking Module (M2)

The parking maneuver aims at positioning the platform close to a roughly planar structure of the environment at a specific location and orientation. This maneuver allows the wheelchair user to approach and stop at specific environment features (table, bed, etc.) or to park away the platform. In order to account for motion errors and unexpected surrounding conditions, the final position and orientation are continuously updated with respect to the reference feature position. The parking strategy is based on a state machine, illustrated in Figure 4, which generates backward and forward motions using the behavioral primitive C3 until the desired pose is reached. A preparatory rotation is performed (primitive C1) before each transition between backward and forward motions. A rotation before stopping improves the orientation accuracy.

3. Door Frame Traversal Module M3

Traversing narrow passages represents a challenge for human and robotic wheelchair controllers. Many previous systems based on commercial wheelchairs have not demonstrated the ability to pass reliably, in autonomous mode, through standard doors less than 1 meter wide (e.g. the system of [37] has shown such a capability with the help of the user only; the system of Montesano [15] mentions such maneuvers without commenting on their performance.)

**Table 1:** Relation between task-oriented modules and primitive behaviors

**User co commands**	**Agents**	**C1**	**C2**	**C3**
Wall following	M1	*yes*	*no*	*yes*
Parking	M2	*yes*	*no*	*Yes*
Passing through narrow passage	M3	*yes*	*yes*	*No*

Successful execution requires motion accuracy, adaptability to unexpected configurations, and deadlock avoidance. Passage traversal is performed, at each execution time, in three steps: (1) locating precisely the passage with respect to the platform, (2) positioning (or and updating) a sequence of waypoints through the passage, and (3) invoking behavior primitive C2 with the assistance of the obstacle avoidance module.

**Figure 5:**
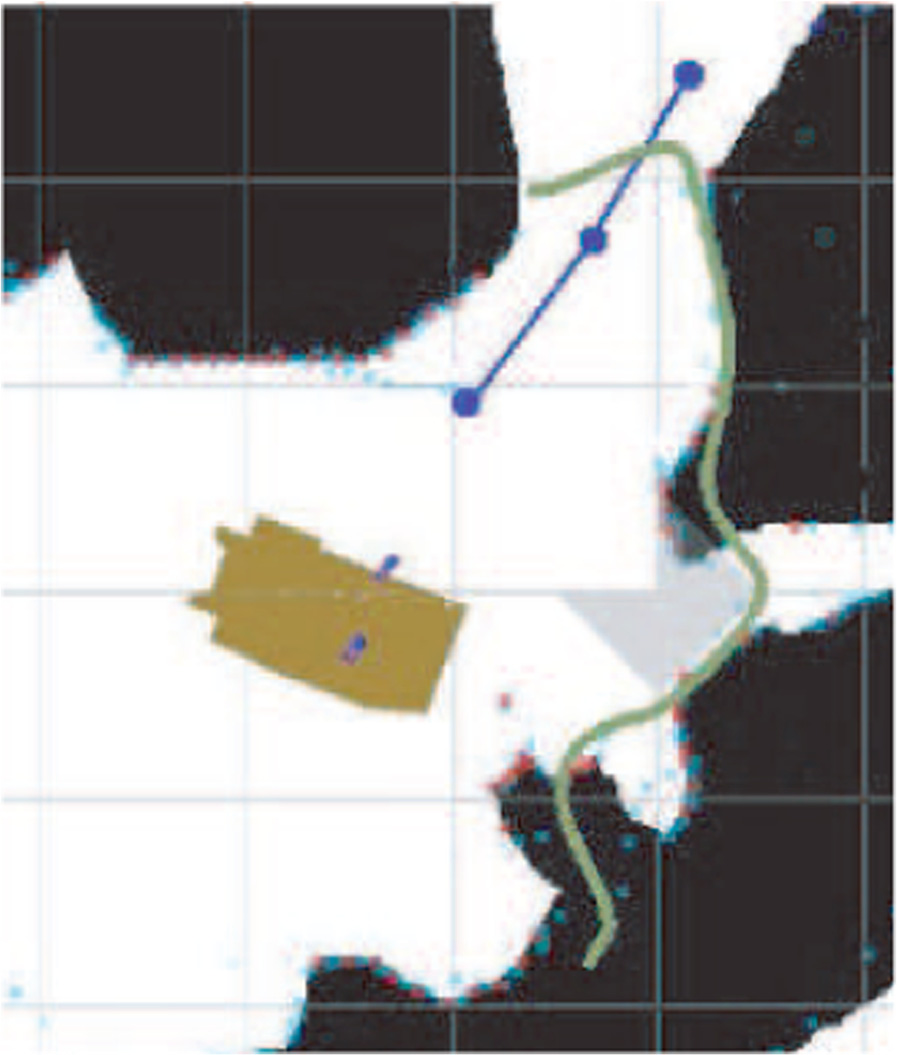
Example of narrow passage and waypoint sequence determination (blue curve).

**Figure 6:**
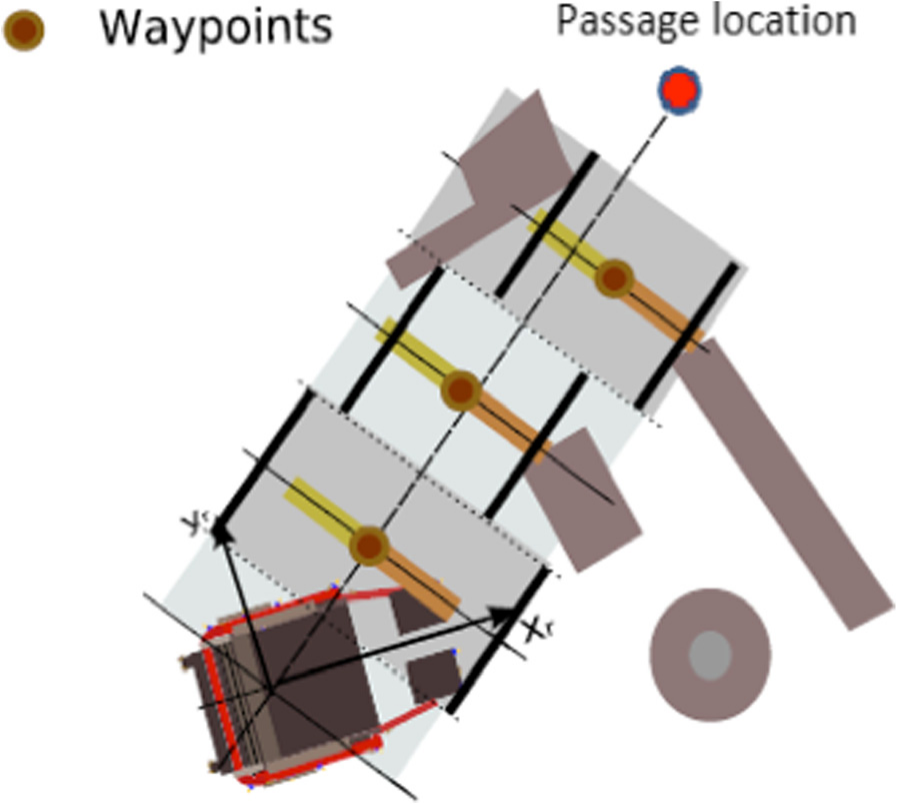
Computation of the waypoint sequence used for narrow door passage.

As illustrated in Figure 5, after each map updating, the location of narrow passages is determined through a low-frequency analysis of the surrounding environment, eliminating locations that are incompatible with the platform geometry. Figure 6 illustrates an example of low-frequency approximation of the immediate environment and the waypoint sequence to traverse the selected passage.

In order to obtain the waypoint sequence, a traversal direction is first established by joining the robot gyration center to the detected passage location. Along that direction, waypoints are defined uniformly at multiples of the platform length. Based upon updated range information, the waypoints are chosen as midpoints between environment left and right limits as shown in Figure 6. In order to ensure that generated waypoints remain close to the traversal line, waypoints are not allowed to get further from that line than half the platform width. Passage traversal is then executed by invoking C2 and C1 primitives with the assistance of the collision avoidance module.

### Interaction system

**Figure 7:**
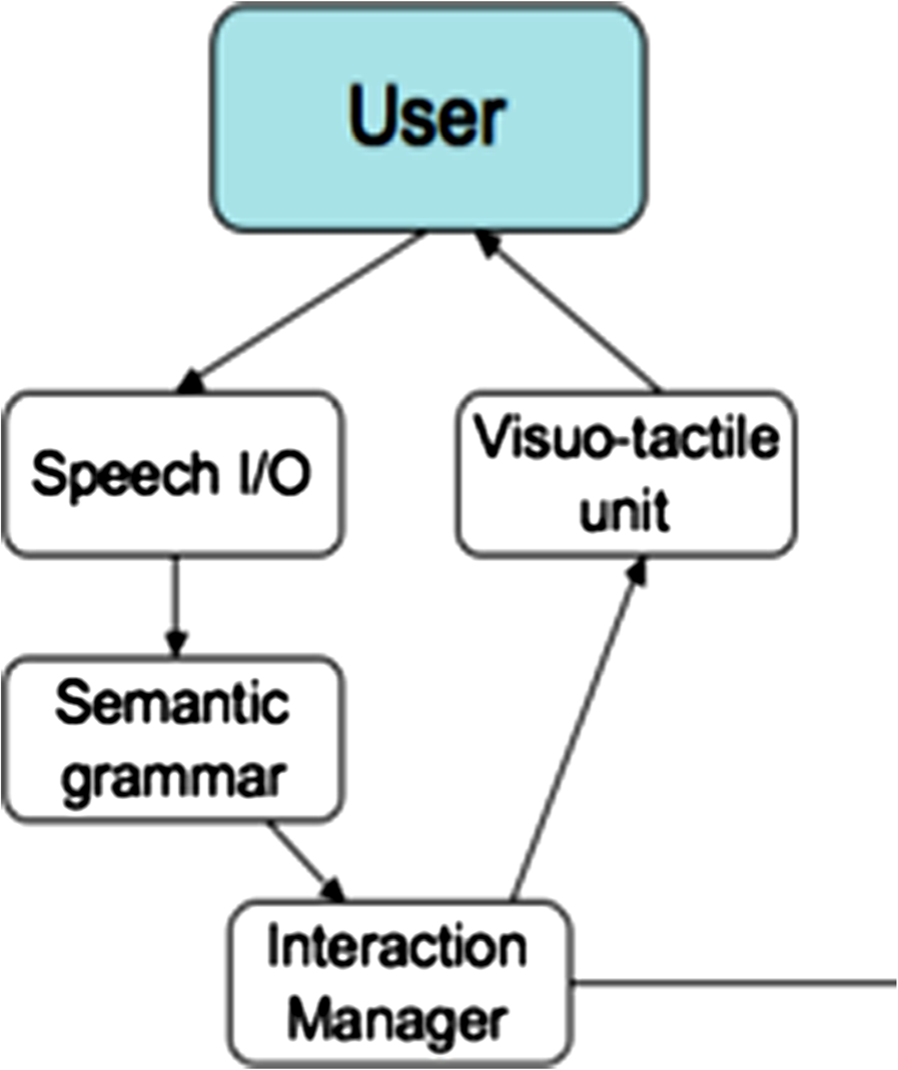
Overview of the interaction architecture.

Direct interaction between the user and the intelligent wheelchair occurs primarily through two modalities. Speech commands are issued by the user, and received by the wheelchair’s onboard processor. In return, the intelligent systems provides visual feedback on the touchscreen, informing the user of what has been understood, and what actions have been selected in response. Speech commands have been used as the primary mode of input in a few wheelchair systems to date [3]. The use of speech is convenient for a large proportion of the target population. However in many systems, vocal interactions were found to be subject to significant failure rates due to the noise and ambiguity inherent in speech-based communication. To overcome this problem, our system combines high-quality commercial speech recognition software with a number of artificial intelligent techniques designed to track and reduce linguistic and semantic ambiguity. The primary innovation is in the particular combination of semantic analysis, probabilistic tracking, and planning algorithms. The empirical results presented in the latter section confirm that this is a successful approach to achieve robust speech-based human-robot interaction. Figure 7 outlines the main components of the interaction system.

#### Speech recognition module

Speech recognition is achieved through the commercially available Dragon NaturallySpeaking (version 9). In earlier work, we considered different publically available automatic speech recognition packages, however the word and sentence error-rates were substantially higher [38]. The other advantage of our chosen system is its ability to robustly handle multiple languages. This is particularly useful in a community such as Montreal, where both English and French are prevalent in the target population. Speech recognition is usually more effective when speakers use their native language. All results reported below are for English language test subjects. However the full interaction system was developed and tested in preliminary experiments with both French and English subjects.

Before the experiment begins, each user is asked to undergo the standard speaker customization procedure suggested by the NaturallySpeaking package. This involves reading a set list of sentences and requires about 10 minutes.

In the current implementation, speech recognition is operated in a touch-to-speak mode, meaning that the user is required to hold down a button while it issues a speech command. In the future, this could be alleviated with software modifications through the NaturallySpeaking SDK, for example by incorporating a keyword to initiate the dialogue. In general, it is not trivial to develop speech interfaces that are effective without explicit conversation initiation, due to the potential for interference form other conversations (either by the main user, or by bystanders).

We impose no restrictions on the vocabulary allowed to interact with the intelligent wheelchair. The user is free to use whatever words and expressions seem best suited to the task. Thus there is no need to memorize specific commands, or undergo further training before using the system. We will see below how the system is customized, through supervised learning, to match certain words to specific actions.

#### Semantic grammar

The output of the speech recognizer is processed by a natural language parser, which extracts syntactic and semantic information from the string of words. The primary role of the grammar is to constraint the output space of the speech interface. The framework we use for natural language parsing is that of Combinatory Categorial Grammars (CCG) [39], for which an open-source implementation is freely available [40].

The grammatical parser transforms the string of words into a logical representation. Thus sentences with different grammatical constructions can be mapped to the same representation. Similarly, different words with identical semantic meanings can also be mapped to each other. This component plays an important role in reducing the large space of sentences that can be recognized to a more compact set of observations.

The grammatical parser requires the designer to provide a set of parsing rules, defining the mapping between natural language and logical form. We provided an initial set of such rules based on domain knowledge. The set of rules was then manually incremented following preliminary evaluation of the interaction system [38].

#### Tracking the dialogue state

We assume the user’s requests can be matched to a finite set of pre-defined dialogue states, each corresponding to a high-level command to the navigation system. The set of dialogue states is constructed using background knowledge of the task domain. For the Wheelchair Skills Test domain, which is the subject of the evaluation described below, we used a set of 27 dialogue states, listed in Table 2. Each dialogue states is executed using one or several primitive behaviors specified in Table 1.

**Table 2:** States of the dialogue manager for the Wheelchair Skill Test domain

	**States**
1	Avoid obstacle
2	Drive slowly backward
3	Drive slowly forward
4	Drive slowly one meter backward
5	Drive slowly two meters backward
6	Go down ridge
7	Move joystick away
8	Move joystick back
9	Roll forward
10	Set speed to fast
11	Set speed to medium
12	Tilt seat backward
13	Turn controller off
14	Turn controller on
15	Turn ninety degrees (left or right)
16	Drive fast backward
17	Drive fast forward
18	Tilt seat forward
19	Drive one meter forward
20	Drive two meters forward
21	Drive backward
22	Align to wall
23	Stop
24	Veer (left or right)
25	Turn a little (to the left or to the right)
26	Pass through door
27	Parking (to the left or to the right)

The semantic grammar outputs a matching set of observations (one per state). It would be most straight-forward to assume that the observation selected by the semantic grammar provides full state information, yet this may be inaccurate, in particular in cases where the speech recognition suffered from word substitution errors, as is relatively common. To provide additional robustness, we assume that the observations are generated probabilistically.

Let *S* be the set of states corresponding to the set of core commands, and *O* be the set of observations providing information about the state. We assume that states evolve according to a Markovian process: *Pr(s*_*t*_*| s*_*t-1*_*),* and that observations are generated probabilistically: *Pr (z*_*t*_*| s*_*t*_*)*. We can compute a posterior over the state: *Pr (s*_*t*_*| z*_*t*_*, …, z*_*1*_*, s*_*0*_*)*, using Bayes rule [41]. We assume initially a set of hand-crafter models for the state transition and observation generation processes. However these models are further refined using machine learning techniques, as detailed below.

#### Learning user models

The state-tracking module relies on having probabilistic models describing how states change over time, and how observations are emitted as a function of state. One of the core challenges of applying such probabilistic methods to human-robot interaction domains lies in acquiring accurate models of these processes. In general, these models can be derived either from expert knowledge, or else directly from data using machine learning techniques. In our system, we combine both. The expert knowledge allows us to achieve reasonable baseline performance without any data; once data is available, we can improve the model to better reflect the reality.

Different learning techniques can be applied to estimate such probabilistic models. We focus on Bayesian learning, which allows us to combine the expert knowledge and the data collected in a coherent framework. We assume the transition parameters, *Pr(s*_*t*_*| s*_*t-1*_*),* and emission parameters, *Pr(z*_*t*_*| s*_*t*_*),* are distributed according to a Dirichlet distribution. Parameters of the Dirichlet are initially set based on expert knowledge. A posterior over the probabilistic distribution is computed by estimating the Dirichlet parameters based on observed trajectories [42,43]. We assume the trajectories are hand-labeled to allow closed-form estimation of the Dirichlet parameters.

#### Robust action selection

One of the principal challenges of managing the interaction between the user and the intelligent wheelchair consists in deciding when to pass on the command identified during state tracking, and when to seek additional information to clarify confusing or incomplete commands. To take full advantage of the probabilistic state tracking component, we integrate a probabilistic decision-making engine based on Partially Observable Markov Decision Processes (POMDP) [44]. In addition to the sets of states and observations outlined above, we also consider a set of actions, one per command (state) plus four more clarification queries that prompt the user for additional information. One is a general query (e.g. “*Please repeat your command”)*, while the other three queries probe the user for information of different types (e.g. “*Please clarify in what direction you would like to move.”)*. We assume a cost function that minimizes the number of incorrect actions and the number of unnecessary queries. Full parameterization of the POMDP model for the Wheelchair Skills Test domain is available online [45]. Optimal sequences of actions are selected via point-based approximate dynamic programming over this model [46].

#### Displaying feedback

Whenever the POMDP model issues an action command or a query, a message is displayed on the onboard screen. Perhaps the simplest of all modules, the one controlling this feedback mechanism is nevertheless extremely useful. Its primary purpose is to inform the user that the system is indeed listening and responding to vocal commands. A possibly more subtle effect of the feedback module is to continuously train the user to speak in a way that the wheelchair understands more easily. Indeed, when the wheelchair issues a query, it proposes three actions; many users tend to read these actions out loud exactly as they appear on the screen, instead of choosing more complicated and unusual phrases. Furthermore, since the user knows when the chair understood the command properly, s/he can learn which words or voice tones work best, and conversely, which ones are more likely to result in a misunderstanding.

### Experimental method

The intelligent wheelchair has been put through a standardized validation process, called the Wheelchair Skill Test (WST) [33,47]. The WST version 4.1 is composed of a series of powered wheelchair skills representing the most common situations that WC users might meet in real life situations. Of the many tests available in the literature to characterize wheelchair use [48], the WST is the only one that has been rigorously tested for both validation and reliability [49]. While the original Wheelchair Skill Test, originally designed for users using a standard manual joystick to control their wheelchair, it has been adapted to the validation of robotic wheelchairs (RWST) by selecting 15 maneuvers that can potentially benefit from the adjunction of autonomous navigation abilities as shown in Table 3. The criteria to include skills from the WST into the RWST was whether the skill required any aspects of the intelligent system. Skills that were accomplished the same way with and without the intelligent system were left out, in the interest of time (e.g. “Turns controller on and off”).

**Table 3:** Wheelchair skills included in the Robotic Wheelchair Skill Test (RWST)

**WST 4.1 ID**	**RWST ID**	**Powered wheelchair skills**
8	1	Rolls forward 10 meters
10	2	Rolls backward 5 meters
11	3	Turns left/right in forward propulsion
12	4	Turns left/right in backward propulsion
13	5	Turns left/right 180 degrees
14	6	Lateral manoeuvers (parking)
15	7	Gets through hinged door
20	8	Rolls 100 meters through hallway
21	9	Avoids mobile obstacles
22	10	Ascend 5-degree right incline
23	11	Descend 5-degree right incline
24	12	Ascend 10-degree right incline
25	13	Descend 10-degree right incline
26	14	Rolls on lateral inclined incline
27	15	Rolls 2 meters on gravel

The test environment was built at Centre de Réadaptation Lucie-Brureau (CRLB) in Montreal (Quebec, Canada) to conform to the full specifications of the WST 4.1 protocol, and is used for regular clinical activities conducted at the CRLB, including training and evaluation of conventional and (non-intelligent) powered wheelchairs. It is worth mentioning that the immediate environments for high-level powered wheelchair skills, such as lateral manoeuvring (parking), getting through hinged door, ascending/descending incline, etc., are highly restrained in space, thus represent a real challenge for a robotic wheelchair.

Seventeen individuals participated in the validation experiments. Eight of them were not wheelchair users (i.e. non-users with no physical impairment) and nine of them were regular WC-users. The non-users were recruited from professionals in the rehabilitation field. They were seven occupational therapists and one technician with 10.8 ± 7 years of experience (range 5–25 years) in the evaluation and prescription of motorized WC. Besides participating as subjects, they were uninvolved in the present project. The WC-users were composed of seven men and two women, aged 57.9 ± 19.0 (range 31–85 years old), who suffer from different health conditions: three from multiple sclerosis, three from spinal cord injury, two from arthritis, and one from stroke. All WC-users actively employ powered wheelchairs for their daily displacements, with an average of 6.8 ± 2.6 years of driving experience (range 2–17 years).

Regular WC-users were asked to execute all powered wheelchair skills as described in Table 3 in two experiments: the first experiment using the standard manual joystick (QTRONIX) with the conventional control mode (i.e. without autonomous maneuver execution), and the second experiment using vocal commands and automated command execution.

Non-users were assessed with those two modes, as well as two additional ones (both including automated command execution mode), one using a multi-function game joystick (Saitek AV8R-01, continuous type of interface) and the other using a computer keyboard (discrete type), for comparison purposes with the standard manual joystick and the vocal interface. Control of the wheelchair via these two interfaces required a combination of inputs. Using the game joystick: linear and rotational motions were commanded through the two analog axes on the joystick; high-level operation such as pass-door, parking, wall-following, straight-line motion were commanded by buttons on the joystick (there are 8 buttons of the joystick, each of them is assigned for a specific operation). Using the keyboard: linear and rotational motions were commanded through the arrow buttons on the keyboard; a specific keyboard button was assigned for each high-level operation (e.g. button D for the pass-door operation, button P for parking, button W for wall following, button R for rectilinear motion.) A combination of commands was necessary for some more complex commands, for example, the operation parking to the right using the joystick can be commanded by bending the stick to the right and then clicking on the specifically assigned button on the joystick, while the same operation can be performed on the keyboard by hitting the P button followed by hitting the right- arrow button.

## Results

### Navigation performance analysis

**Figure 8:**
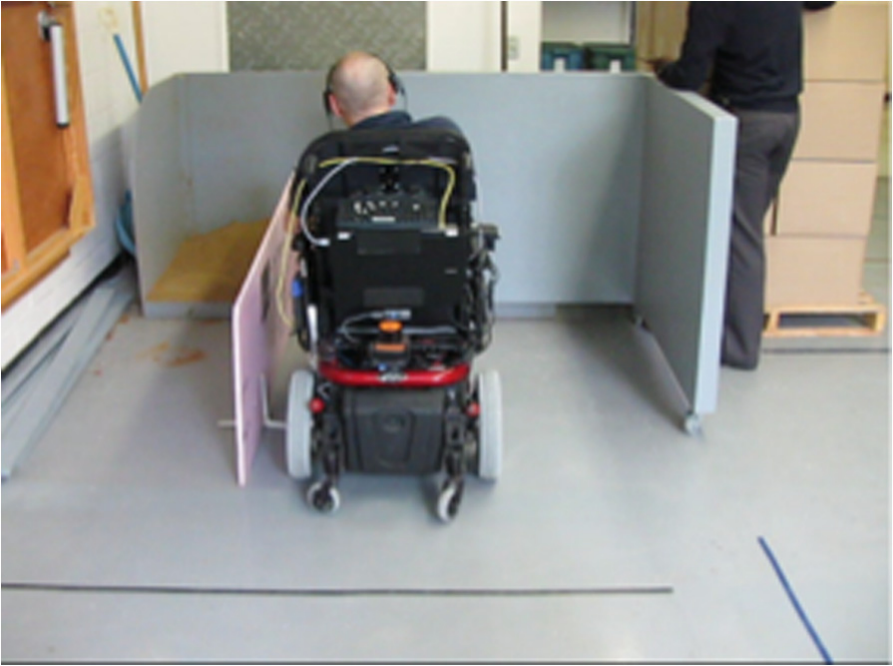
Example of maneuver executions: lateral maneuver (parking).

**Figure 9:**
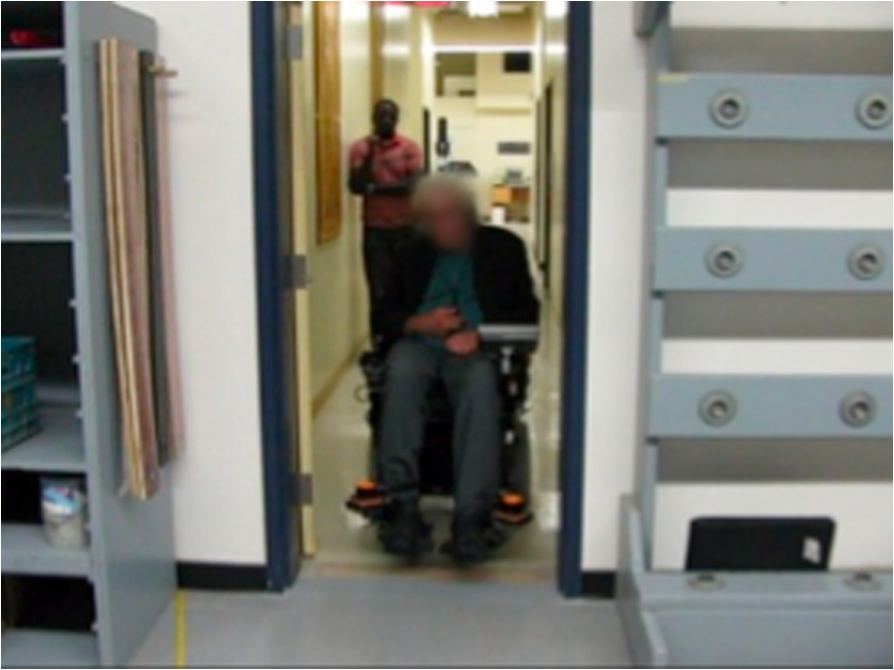
Example of maneuver executions: door frame traversal.

**Figure 10:**
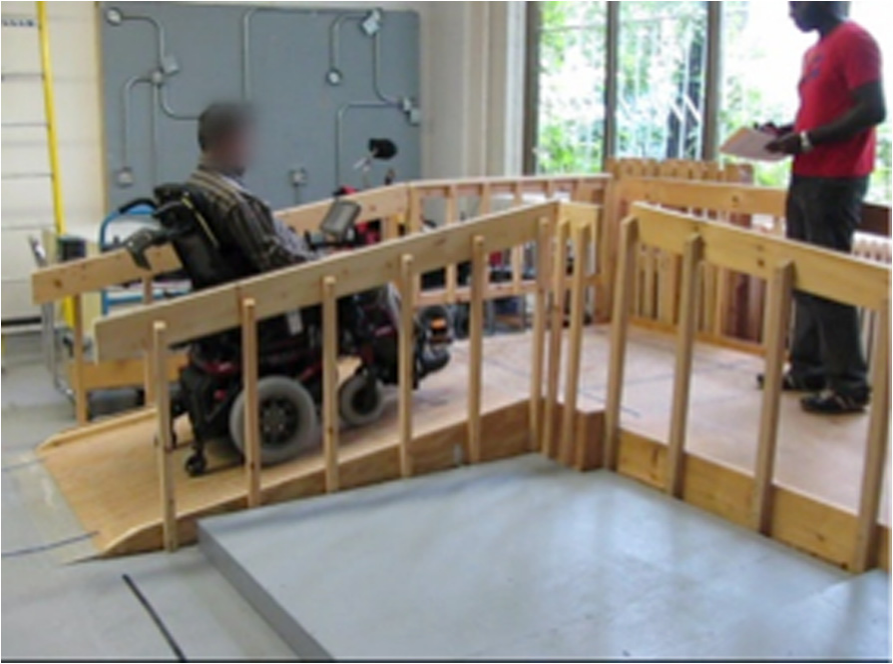
Example of maneuver executions: slope climbing.

**Figure 11:**
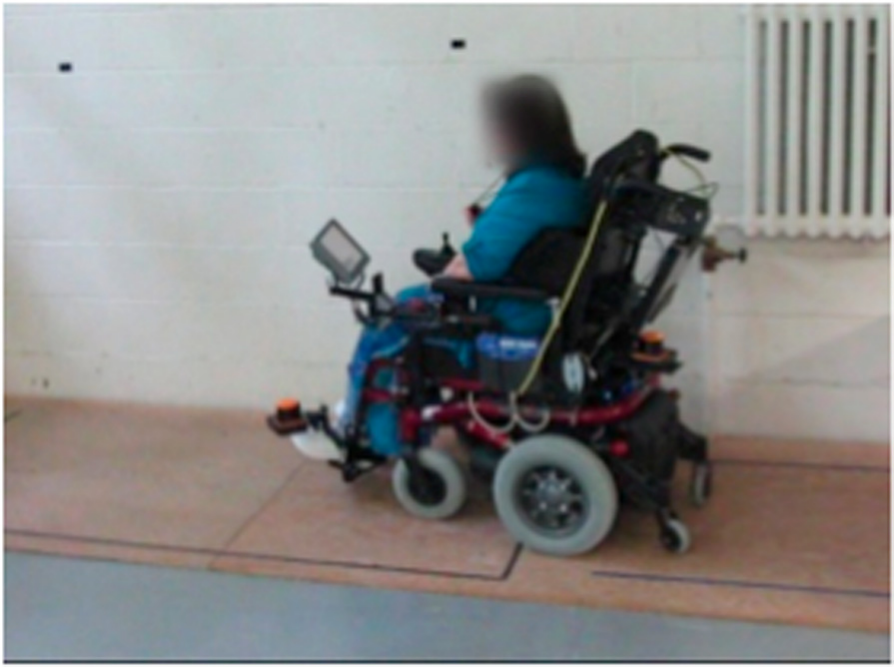
Example of maneuver executions: motion on incline.

**Figure 12:**
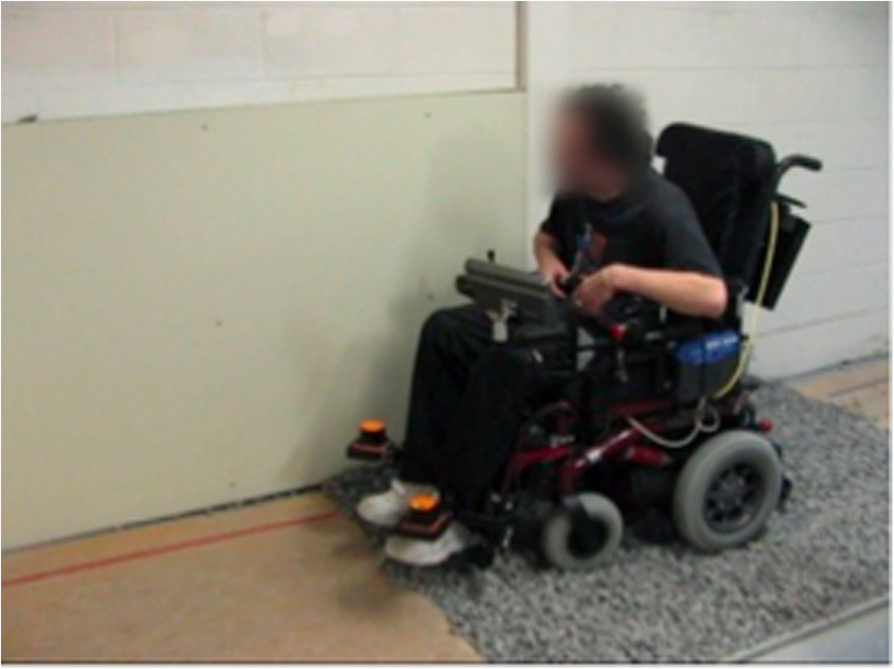
Example of maneuver executions: motion on gravel.

**Figure 13:**
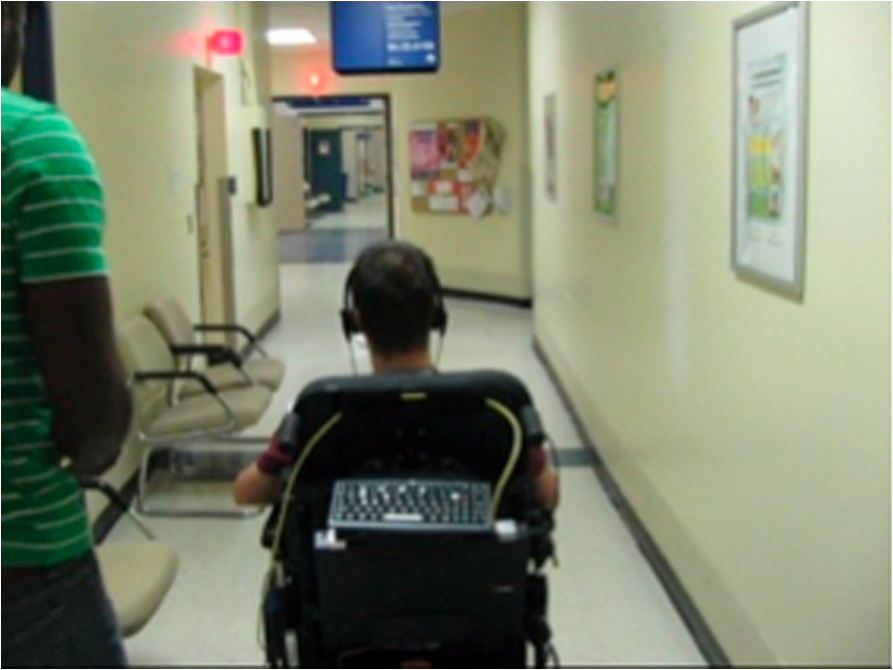
Example of maneuver executions: motion through hallway.

Figures 8, 9, 10, 11, 12, and 13 shows examples of various maneuver executions. Quantitative performance results for the 15 skills in the autonomous (i.e. vocal interface) and standard control (i.e. joystick) modes are compared in our results below.

Table 4 provides measurements of average speeds and minimal distances to obstacles for various maneuvers in the automated command execution mode. We can see the dependency between the operating speed and the minimal distance to obstacles, which is directly related to the speed adaptation of the motion assistance module. Task executions in spatially constrained environments (measured by the average minimal distance to obstacles), such as parking and motions on incline (0.14 m and 0.19 m, respectively, of minimal distance to obstacles) were executed with a low speed (0.15 m/s and 0.13 m/s), while on-floor displacements (straight line moves and turns), usually executed in a relatively obstacle-free space (0.44 m and 0.42 m of minimal distance to obstacle) move with a higher speeds (0.33 m/s and 0.23 rad/s, respectively). Task execution in less constrained spaces, such rolling through hallway or getting through the door (0.27 m and 0.24 m of minimal distance to obstacle) were carried out with a speed of 0.31 m/s and 0.33 m/s, respectively. It is worth mentioning that when getting through the door, only part of the operation were executed in the constrained space (when the wheelchair actually passed through the door frame), the remaining part (the beginning and the end) were executed in relatively open space. This dependency between speed and minimal distance to obstacles confirms the ability of the navigation system to regulate motion speed according to various spatial contexts.

**Table 4:** Dependency between moving speed and minimal distance to obstacles (all subjects, n = 17)

**Powered WC skills**	**Measured linear speed (m/s)**	**Measured angular speed (rad/s)**	**Minimal distance to obstacles (m)**
Rolls forward 10 meters, Rolls backward 5 meters	0.33	0.09	0.44
Turns left/right 180 degrees	0.06	0.23	0.42
Ascends 5-degree right incline,	0.13	0.12	0.19
Descends 5-degree right incline,			
Ascends 10-degree right incline,			
Descends 10-degree right incline			
Lateral maneuvers (parking)	0.15	0.13	0.14
Gets through hinged door	0.33	0.10	0.24
Rolls 100 meters through hallway	0.31	0.11	0.27

In the navigation and task execution context, the intelligent wheelchair moves at an average of 0.26 m/s in an environment with 0.28 m of minimal average proximity distance to obstacles. These results are to be compared to the system in Montesano *et al.,*[15], characterized with an average speed of 0.13 m/s and 0.77 m proximity distance, and the system in Sgouros [50] with 0.20 m/s speed and roughly the same proximity distance as in our experiments. This suggests that the system we have developed provides improved navigation efficiency in constrained spaces. It is worth noting that the shape of the wheelchair has an effect on its ability to navigate through tight spaces. The wheelchair can reliably pass through a standard door of 86 cm (34”), compared to its width of 68 cm (27”). Passing through narrower doors was not tested.

Encounters with dynamic objects, such as moving persons or other vehicles (wheelchairs) were tested in the *Rolls 100 meters through hallway* skill, where the examiner tested the reaction of the IPW by presenting himself as a moving person. Other dynamic objects were occasionally encountered during other skills; in some cases, the examiner asked the user to pause the experiment until the dynamic objects had moved, in other cases (where the disturbance was minor), the experiment was allowed to continue.

Collisions and object grazings occurred occasionally during experiments. Both types of events indicate that the wheelchair is in physical contact with an obstacle; object grazings are very minor contact and do not impede the conduct of an operation, whereas collisions may impede the conduct of an event (e.g. require a change in trajectory). No serious collisions (e.g. causing concern for the safety of the subject) were recorded. In average, we recorded 4.7 collisions and 1.8 grazings per kilometer of running distance, compared to 17 collisions per kilometer from Montesano *et al*. [15].

The main causes of contacts (collisions/grazings) between the IPW and objects during operations were the following:

1. Misalignment of laser sensors

Laser sensors on the IPW were calibrated before all testing sessions. The calibration process allows object location around IPW to be determined precisely. Instantaneous motions of IPW (speed, acceleration) are generated based on this information to achieve a specific task. Misalignment of laser sensors provides erroneous object positions, resulting in contacts between IPW and objects. Misalignment of front lasers was mainly caused by the user’s feet (two front lasers are positioned close to user’s feet).

2. Reflective surfaces (such as metallic bodies)

Several contacts were observed during the IPW navigation through corridor where some wall corners are protected by aluminum plates.

3. Objects in the dead zone of laser sensing

This usually occurs during highly restrained space operations such as passing through a door, where nearby objects enter the blind spot between laser sensors (e.g. on the side). Contact with undetected objects also occurred when descending the right incline, where difficulties in dynamic control, combined with the presence of close-range objects, caused a number of observed contacts.

Another limitation of the current platform is the lack of vertical laser sensing, which could lead to collision with objects at varying heights (e.g. tables, parking gates), though this was not observed in the RWST sessions. The addition of sonar sensors may help alleviate many of these problems, especially the detection of reflective surfaces, and the minimization of dead zones. Alternately, many such obstacles could be avoided through the use of vocal commands, requesting that the wheelchair stop.

### Performance scores on robotic wheelchair skill tests (RWST)

Each maneuver of the RWST is scored in terms of performance and safety. In the conventional mode, the performance score measures the capacity of the user, with the use of a manual joystick and without the help of autonomous navigation modules, to execute the task successfully or not. In the autonomous mode, it measures the capacity with which the user, with the help of the autonomous navigation modules, coupled with one of the interfaces (vocal, joystick, keyboard), is able to carry the task successfully. The user had to be able to execute a task on the first or second try to satisfy the performance measure. The safety score measures the occurrence of collisions, environment grazings, or unpredictable behaviors during task execution. If the examiner had to intervene to stop the movement of the wheelchair, then the task was judged as a fail for both safety and performance. Any task that is judged unsafe automatically is judged to fail the performance also (so the performance score isalways lower than, or equal to, the safety score). Tables 5 and 6 contain average scores for the non- user and WC-user populations respectively in each task, while Tables 7 and 8 show the performance (averaged over all operations) of each individual.

**Table 5:** Average scores for non-users (n = 8)

**RWST operation ID**	**Powered WC skills**	**Performance (%)**		**Safety (%)**	
		**Vocal interface**	**Standard joystick**	**Vocal interface**	**Standard joystick**
1	Rolls forward 10m	100	100	100	100
2	Rolls backward 5m	87.5	100	87.5	100
3	Turns left/right in forward propulsion (n = 7)	85.7	100	100	100
4	Turns left/right in backward propulsion	83.3	100	100	100
	Joystick (n = 7); Vocal (n = 6)				
5	Turns left/right 180 degrees	83.3	100	83.3	100
	Vocal (n = 6)				
6	Lateral maneuvres (parking)	100	100	100	100
7	Gets through hinged door	100	100	100	100
8	Rolls 100 meters in hallway	100	100	100	100
	Vocal (n = 7)				
9	Avoids mobile obstacles	100	100	100	100
	Vocal (n = 7)				
10	Ascends 5-degree right incline	100	100	100	100
11	Descends 5-degree right incline	100	100	100	100
12	Ascends 10-degree right incline	100	100	100	100
13	Descends 10-degree right incline	100	100	100	100
14	Rolls on laterally inclined incline	100	100	100	100
	Vocal (n = 7)				
15	Rolls 2 meters on gravel	100	100	100	100
Average	All 15 tasks	96.0	100	98.1	100

**Table 6:** Average scores for WC-users (n = 9)

**RWST operation ID**	**Powered WC skills**	**Performance (%)**		**Safety (%)**	
		**Vocal interface**	**Standard joystick**	**Vocal interface**	**Standard joystick**
1	Rolls forward 10m	100	100	100	100
2	Rolls backward 5m	100	100	100	100
3	Turns left/right in forward propulsion	88.9	100	100	100
4	Turns left/right in backward propulsion	88.9	100	88.9	100
5	Turns left/right 180 degrees	66.7	100	88.9	100
6	Lateral maneuvers (parking)	100	100	100	100
7	Gets through hinged door	100	100	100	100
8	Rolls 100 meters in hallway	77.8	100	77.8	100
9	Avoids mobile obstacles	100	100	100	100
10	Ascends 5-degree right incline	100	100	100	100
11	Descends 5-degree right incline	100	100	100	100
12	Ascends 10-degree right incline	100	100	100	100
13	Descends 10-degree right incline	100	100	100	100
14	Rolls on laterally inclined incline	100	100	100	100
15	Rolls 2 meters on gravel	100	100	100	100
Average	All 15 tasks	94.8	100	97.0	100

**Table 7:** Individual scores for the 15 tasks performed by non-users (n=8)

**Subjects**	**Performance (%)**		**Safety (%)**	
	**Vocal interface**	**Standard joystick**	**Vocal interface**	**Standard joystick**
S1	93.3	100	100	100
S2	100 (n = 12)	100 (n = 13)	100 (n = 12)	100 (n = 13)
S3	100 (n = 13)	100	100 (n = 13)	100
S4	92.9 (n = 14)	100	100 (n = 14)	100
S5	100 (n = 13)	100	100 (n = 13)	100
S6	86.7	100	86.7	100
S7	100	100	100	100
S8	100	100	100	100

(Calculated as a ratio between the number of tasks that were successful, over the number of tasks attempted).

**Table 8:** Individual scores for the 15 tasks performed by WC-users (n = 9)

**Subjects**	**Performance (%)**		**Safety (%)**	
	**Vocal interface**	**Standard joystick**	**Vocal interface**	**Standard joystick**
H1	73.3	100	80	100
H2	100	100	100	100
H3	100	100	100	100
H4	100	100	100	100
H5	93.3	100	100	100
H6	100	100	100	100
H7	100	100	100	100
H8	93.3	100	100	100
H9	93.3	100	93.3	100

(Calculated as a ratio between the number of tasks that were successful, over the number of tasks attempted).

As shown in Tables 5, 6, 7, 8, the performance of the two groups of individuals in automatic mode is comparable. These results show an average decrease of 4% in performance score and 3% in safety score, compared to the scores obtained with the conventional driving mode. We note that all subjects included in the experiment are proficient at controlling the wheelchair in conventional joystick mode, thus what we are establishing here is that they can achieve a near-similar level of performance with the vocal interface and intelligent command mode. It should be noted that the regular users had over 6 years of wheelchair driving experience, compared to approximately one half-hour of training with the autonomous mode.

### Comparison of user interfaces

Detailed performance analysis of the dialogue interface is provided in Pineau et al. [51]. Summary statistics from this analysis are presented in Table 9, including the average number of commands to complete the RWST, the average word error rate, as well as the average number of clarification queries, number of correct actions, and number of incorrect actions used during the RWST. These statistics were obtained by manually labeling the interactions from video recordings. These results in Table 9 show that while the per-word accuracy is far from perfect (14% word error rate for non-users; 19% for users), subjects are able to successfully accomplish tasks with few clarifying queries (19 for non-users; 21 for users) and few errors (5 incorrect actions in 168 commands for non-users; 5 incorrect actions in 130 commands for users).

**Table 9:** Performance of the interaction manager using the vocal interface

	**Non-users**	**WC-users**
Number of commands	168 ± 26	130 ± 14
Word error rate	14 ± 5	19 ± 7
Number of queries	19 ± 7	21 ± 9
Number of correct actions	144 ± 25	105 ± 11
Number of incorrect actions	5 ± 2	5 ± 2

As mentioned previously, non-users tested two additional interface modalities (the game joystick and the keyboard). Table 10 reports average score comparisons in the case of non-wheelchair users for the three input modes; the same underlying navigation architecture is used in all three cases. As expected, the vocal interface scores are slightly lower than the others in terms of performance and safety. It is worth noting that with the keyboard interface, non-users achieve a performance score of 99% and a safety score of 100%. These results suggest that the intelligent system itself performs very well, but that the use of the vocal interface introduces problems. However this was not tested with WC-users, and furthermore, the keyboard interface may be difficult to use for some wheelchair-users.

**Table 10:** RWST performance comparison for non-users

**Interface**	**Performance (%)**	**Safety (%)**
Vocal (n = 112)	96.0	98.1
Game Joystick (n = 105)	98.1	99
Keyboard (n = 117)	99.1	100

(Some subjects did not complete all tasks, so the number of instances per interface varies slightly).

The results presented in Table 10 are consistent with the statistics on collisions and grazing provided in Table 11. The slight increase in collisions and grazing with the vocal interface can be explained by the presence of a substantial delay of about 2 seconds in command executions compared to a delay of approximately 0.5 second for the other interfaces. This delay was mostly caused by software limitations in the integration of speech recognition software. It is believed that a delay reduction would bring the vocal interface performance to the same level as the other interfaces. There is could be achieved with appropriate software configuration (it was not possible at the time of the experiments due to lack of the right software development kit).

**Table 11:** Collision and grazing statistics

**Interface**	**Average number of collisions per test subject**	**Average number of Grazing per test subject**
Non-users, Vocal interface (n = 8)	1.2	0.9
Non-users, Game Joystick (n = 7)	0.7	0.1
Non-users, Keyboard (n = 8)	0.1	0.1
WC-users,Vocal interface (n = 9)	0.9	0.3

## Conclusion

This paper provides a comprehensive overview of the design and validation of a new intelligent wheelchair platform. First, we present navigation architecture for control in constrained spaces; this has been shown to achieve greater speeds than previous systems in tight spots. Second, we describe a decision-theoretic approach for achieving speech-based control of the intelligent wheelchair. This component requires very little training, and can be used to achieve reasonable performance, though there are some weaknesses that we discuss more extensively below.

One of the main innovations of the work presented here is the focus on a functional relevant domain and evaluation metric that has been validated [47], in addition to the traditional robotic metrics. The RWST requires some investment in terms of infrastructure and space, but these are by no means unreasonable. By performing this evaluation, we can provide solid evidence on the potential impact of the robotic technology; this is an important step towards gaining acceptance in the clinical community.

Overall, with 17 test subjects, 32 complete RWST sessions, 25 total hours of testing, and 9 kilometers of total running distance, the platform tested in these experiments is among the most experimentally validated robotic wheelchairs in realistic contexts (compared to similar systems reported recently: Montesano et al. [15], Urdiales et al. [12], and Ju et al. [31]).

Yet despite these highly promising results, the intelligent wheelchair still falls short of the performance measured with conventional (joystick) control, where all subjects were able to achieve 100% performance and safety scores on the RWST tasks.

There are some technological limitations in the current system. The navigation modules focus on a local representation of the environment; this is sufficient for small domains, providing flexibility and low computational burden. However this is insufficient for tackling navigation in larger spaces. Fortunately, this problem has been extensively studied in the robotics literature, and we expect that many existing technologies can be leveraged. The intelligent wheelchair is already able to perform a number of tasks that were not necessary for the WST (and thus were not described in this paper), including navigating in narrow spaces, and following another person who is walking along.

The development of the interface also poses some interesting challenges. To date, our investigation has focused primarily on the development of the vocal interface. This input mode tends to be most accessible for many users, but is not without difficulties. We have overcome some of the problems related to errors in the recognition through a combination of Bayesian filtering and machine learning. Some of the remaining performance gap, compared to manual control modes, is due to the longer time delay required to process the input. This delay seems also responsible for the increase collisions in tight spot requiring sharp turns. There are simple software solutions to this problem that were not available at the time of conducting our experiments. Another factor that could influence the difference between the autonomous vs. standard mode is that the dialogues used with the vocal command did not include many choices as to the amount of turning (i.e. number of degrees). Again, this was particularly evident in the tasks requiring precise turns to stay within the required surface area. In the longer-term, as we prepare to deploy our intelligent WC in richer environments, further issues such as out-of-vocabulary commands will have be addressed. We are developing techniques that can individually tailor the dialogue system to a particular user in an effort to address this challenge.

Finally, the results we present also suggest that there is a need for more challenging evaluation metrics to establish the usefulness of intelligent wheelchairs, since all subjects were able to achieve perfect performance and safety scores in the RWST using conventional control. It would be useful to identify a set of tasks that are particularly challenging for many wheelchair users, and use these to define a new instrument for characterizing the performance and safety of wheelchair users.

## Consent

Written informed consent was obtained from the patient for publication of this report and any accompanying images.

## Competing interests

The authors declare that they have no competing interests.

## Authors’ contributions

PC, FR, RF and JP conceived of the study and participated in the design of the IPW. PB, SK and HN developed the navigation architecture, implemented it onboard the IPW, participated in the user study with the RWST, and analyzed the data pertaining to the navigation performance of the IPW. AA and JV developed the dialogue architecture, implemented it onboard the IPW, participated in the user study with the RWST, and analyzed the data pertaining to the dialogue system of the IPW. WH, LD, FR and RF developed the experimental protocol for the RWST, and supervised all user experiments. WH, FR and RF carried out the analysis of the RWST data. PB, SK, HN, JV, FR, PC, RF and JP helped draft the manuscript. All authors read and approved the final manuscript.

## Authors’ information

During this study, PB was an MSc candidate at Ecole Polytechnique de Montréal, AA was a PhD candidate at McGill University, SK was a PhD candidate at Ecole Polytechnique de Montréal. WH was an MSc candidate at the Université de Montréal. HN was and continues to be an engineer at Ecole Polytechnique de Montréal. JV was an MSc candidate at McGill University. FR was a researcher at the Institut de réadaptation en déficience physique de Québec; he is now a professor at Université Laval. PC was a professor at Ecole Polytechnique de Montréal, where he is now an adjunct professor. LD and RF continue to be professors at the Université de Montréal. JP is a professor at McGill University.
